# Participation of HIV prevention programs among men who have sex with men in two cities of China—a mixed method study

**DOI:** 10.1186/1471-2458-12-847

**Published:** 2012-10-08

**Authors:** Wei Ma, H Fisher Raymond, Erin C Wilson, Willi McFarland, Hongyan Lu, Xianbin Ding, Rongrong Lu, Xiaoyan Ma, Dongyan Xia, Jing Xu, Xiong He, Liangui Feng, Song Fan, Xuefeng Li, Jiangping Sun, Yujiang Jia, Yiming Shao, Yuhua Ruan, Yan Xiao

**Affiliations:** 1 Shandong University School of Public Health, Jinan, China; 2 San Francisco Department of Public Health, San Francisco, CA, USA; 3 Beijing Municipal Centre for Disease Control and Prevention, Beijing, China; 4 Chongqing Municipal Centre for Disease Control and Prevention, Chongqing, China; 5 State Key Laboratory for Infectious Disease Prevention and Control, and National Centre for AIDS/STD Control and Prevention, Chinese Centre for Disease Control and Prevention, Beijing, China; 6 Institute for Global Health and Department of Preventive Medicine, Vanderbilt University School of Medicine, Nashville, TN, USA

**Keywords:** MSM, HIV prevention programs, Utilization, Participation, China

## Abstract

**Background:**

Although various HIV prevention programs targeting men who have sex with men (MSM) are operating in China, whether and how these programs are being utilized is unclear. This study explores participation of HIV prevention programs and influencing factors among MSM in two cities in China.

**Methods:**

This is a mixed-method study conducted in Beijing and Chongqing. A qualitative study consisting of in-depth interviews with 54 MSM, 11 key informants, and 8 focus group discussions, a cross-sectional survey using respondent-driven sampling among 998 MSM were conducted in 2009 and 2010 respectively to elicit information on MSM’s perception and utilization of HIV prevention programs. Qualitative findings were integrated with quantitative multivariate factors to explain the quantitative findings.

**Results:**

Fifty-six percent of MSM in Chongqing and 75.1% in Beijing ever participated in at least one type of HIV prevention program (P=0.001). Factors related to participation in HIV prevention programs included age, ethnicity, income, HIV risk perception, living with boyfriend, living in urban area, size of MSM social network, having talked about HIV status with partners, and knowing someone who is HIV positive. Reasons why MSM did not participate in HIV prevention programs included logistical concerns like limited time for participation and distance to services; program content and delivery issues such as perceived low quality services and distrust of providers; and, cultural issues like HIV-related stigma and low risk perception.

**Conclusions:**

The study shows that there is much room for improvement in reaching MSM in China. HIV prevention programs targeting MSM in China may need to be more comprehensive and incorporate the cultural, logistic and HIV-related needs of the population in order to effectively reach and affect this population’s risk for HIV.

## Background

Men who have sex with men (MSM) are recognized as being at high risk for HIV infection in the Western world [1,2]. Recently, there have been reports of new or newly identified epidemics of HIV infection among MSM in Asia, Africa and Latin America [3,4]. Sexual transmission accounted for more than 70% of new infections in China. Of these sexually transmitted new infections, MSM accounted for 32.5% in 2009, which is almost a 3-fold increase from 12.2% in 2007 [5,6]. Recent studies have documented dramatic increases in HIV prevalence and alarmingly high HIV incidence among MSM in major Chinese urban areas [7-9]. A national study targeting over 18,000 MSM in 61 cities reported an average HIV prevalence of 4.9%, with prevalence up to 20% in several urban areas [10]. For example, in Chongqing, the largest municipal administrative unit in China with a population of 32 million, HIV prevalence increased from 5.8% in 2005, 10.4% in 2006, 12.5% in 2007 to 16.8% in 2008 among MSM, and HIV incidence is almost 8% [9,11]. The Chinese National Medium and Long-Term Strategic Plan for HIV/AIDS Control and Prevention (1998–2010) identified MSM as a high-risk group for HIV infection [12], however, this population was not a primary target population for HIV prevention until 2004 [13,14]. Although the resulting intervention programs disseminated AIDS information and increased awareness of HIV/AIDS among MSM, many studies have found that the prevalence of risky behaviours such as multiple partners, inconsistent condom use, and not being routinely tested for HIV remained high among this population. These trends suggest that the HIV epidemic is worsening among Chinese MSM, who may soon become the single group most affected by HIV/AIDS in China. Moreover, it is estimated that there are 5–10 million MSM in China [15]. Thus, if HIV transmission is not effectively controlled among this population, China will become the global centre of MSM transmission of HIV.

HIV intervention and prevention programs including distribution of condoms/lubricants, providing voluntary HIV counselling and testing (VCT) and sexually transmitted infections (STIs) testing and treatment have been documented to be effective in reducing high risk behaviours among MSM. In a meta-analytic review of such HIV behavioural interventions for reducing sexual risk behaviour among MSM conducted by Herbst (2005), HIV interventions were associated with a significant decrease in unprotected anal intercourse and number of sexual partners (OR = 0.85,) and with a significant increase in condom use during anal intercourse (OR=1.61) [16]. Johnson (2002) also demonstrated that interventions can promote risk reduction among MSM [17]. In China, existing intervention studies show strong evidence of controlling the HIV/AIDS epidemic through effective behavioural interventions. However, basic HIV prevention services have yet to reach the large majority of MSM in developing countries, which is also the case in China [18]. Homophobia and discrimination have been found to limit access of MSM to prevention services, thus markedly increasing their vulnerability to HIV [19]. Additionally, investment in prevention programs for MSM in China as elsewhere remains inadequate compared to the contribution of male-to-male transmission to the overall burden of the HIV epidemic. For example, most Asian countries have started addressing the HIV needs of MSM, but the coverage seems to be far from the 60-80% level needed to have an effect on the HIV epidemic [20]. Without an investment relative to the risk to MSM for acquiring this disease, HIV rates will continue to climb among MSM and China as a whole.

In 2005, the Chinese Government started to strengthen its intervention efforts among MSM developing national working protocols and guidelines. Various programs were conducted including condom promotion, counselling and testing, peer education and STI services. The third quarter 2007 statistics showed that 88,082 MSM were reached by comprehensive HIV prevention interventions, a coverage of around 8.2 per cent of the MSM population [5]. The most commonly used intervention strategy in the early stage was individual-oriented HIV-related knowledge education and behavioural skills training [18]. Zhang showed that in 2006, there were four models of interventions targeting MSM: health education via communication with friends, peer education, MSM venues intervention, and health counselling. Each of the models had its distinction in terms of population coverage, financing and operational mechanism and each complemented one another [21].

Although various HIV prevention programs targeting MSM are operating in China, the level of utilization of these programs remains unclear in many areas. Steward (2008) showed that MSM were less likely to receive these services than non-MSM even though MSM were more likely to report unprotected sex [22]. Understanding the proportion of MSM who have participated in HIV prevention programs and factors associated with their participation is central to understanding how large the coverage gap among MSM is and identifying characteristics of the MSM who have had no exposure to HIV prevention programs in order to tailor outreach and intervention approaches [23].

In 2009 and 2010, we conducted a mixed methods study in two large cities in China- Chongqing and Beijing- to describe utilization of HIV prevention programs among MSM. The study utilized a respondent driven sampling (RDS) survey to determine the proportion of MSM who have had exposure to such interventions in the past year, to find factors associated with participation in HIV prevention, and to gather qualitative data for exploring MSM’s perceptions and needs for HIV prevention programs.

## Methods

This is a mixed-method study consisting of a RDS survey and a qualitative study conducted in Chongqing and Beijing in 2009 and 2010. Qualitative data were originally collected to inform the development of the survey. Due to the richness of the findings, the team decided to utilize the qualitative data to contextualize findings on HIV prevention participation of Chinese MSM. This study utilized an expansion mixed methods design [24]. Using an expansion design, authors combined qualitative and quantitative methods in order to yield a rich understanding of HIV prevention program participation among Chinese MSM. This approach allowed authors to assess the phenomena of HIV prevention program participation both from the perspective of coverage and to determine facilitators and barriers to program participation that could be intervened upon in the two provinces.

### Participants

In 2009, the qualitative study was conducted to elicit information on MSM’s perception of HIV prevention programs. In-depth interviews with 54 MSM were conducted in the two cities. MSM Participants were purposively sampled to capture maximum variation in age, education and HIV status. We aimed to gather a diverse sample of MSM to capture the variety of views and experiences within this population. Inclusion criteria for in-depth interview participants were: 1) having had sex with men in the last 12 months; 2) having resided in the local area for at least 6 months; 3) 18 and above years of age; and, 4) willing to participate in the study.

Eight focus group discussions were conducted with 52 participants. Eligible participants were: 1) MSM with education level of high school and below; 2) MSM with education level of college and above; 3) MSM aged 40 years old and above; and 4) MSM living with HIV/AIDS. Lastly, we conducted in-depth interviews with service providers, volunteers, and researchers who participate in prevention, care, and research on HIV/AIDS among MSM (N=11). Inclusion criteria for health providers, volunteers and researchers were: 1) having provided HIV/AIDS-related services or having conducted research among MSM; 2) having resided in the locale for at least 6 months; 3) 18 and above years of age; and, 4) willing to participate in the interview. All participants were recruited through referrals from local community leaders, local Centres for Disease Control and Prevention (CDC) researchers, and through outreach visits to venues known to be frequented by MSM, including parks, bars, bath houses, and Internet cafes. The final in-depth interview sample consisted of 5 CDC staff and 29 MSM in Beijing, of which there were 8 outreach workers. In Chongqing, the qualitative sample consisted of 6 CDC staff and 25 MSM, including 1 outreach worker.

The cross-sectional RDS survey was conducted among MSM in 2010 in Chongqing and Beijing. The same inclusion criteria as the in-depth interviews applied, with the addition of having a valid study recruitment coupon. Participants of the survey were recruited using respondent-driven sampling (RDS). Six and ten MSM in Chongqing and Beijing, respectively, were selected to function as recruiter ‘seeds’ and were diverse with respect to the types of venues that they frequented. Seeds were evaluated for their commitment to the goals of the study and motivation to recruit three eligible peers in their social network. Seeds were each asked to recruit up to three participants, who in turn were asked to recruit a subsequent wave of up to three participants, and so on, until our target sample size was reached and equilibrium was achieved on key variables. Each participant was given three recruitment coupons/cards with study information to hand to potential recruits. To keep track of social networks, each card had a number code that connected participants back to the initial seeds. Participants were compensated 30 Yuan (CNY)(about 4.78 US dollars) for their participation in the study, as well as 20 Yuan (CNY)(about 3.18 US dollars) for each eligible participant they recruited who subsequently completed a study interview.

### Data collection

In the qualitative study, information on experiences participating in HIV prevention programs, perception of HIV prevention programs being good or bad, and facilitators of and barriers to participating in HIV prevention programs was elicited. Face to face, in-depth interviews were conducted using a semi-structured interview guide that allowed participants to elaborate on topics of particular interest. The interview guide was developed using theories of behaviour change, findings from literature, the opinions of experts, and with an understanding of the HIV epidemic in China. The guide utilized the Health Belief Model, specifically focusing on key constructs of perceived susceptibility, perceived barriers and benefits, and self efficacy, and the Social Cognitive Theory, which emphasizes interacting and reciprocal relationships between behaviour, personal factors (including cognitions), and environmental influences [25]. The guide was pilot tested by MSM from the community and CDC staff.

Participant interviews lasted approximately one to two hours and were audio-taped if the participant agreed. Interviewers underwent training in interviewing techniques and the interview guide prior to the start of data collection. Focus group discussions were conducted with 6–8 people per group, lasted approximately one to two hours, and were led by one facilitator trained by the research team. An assistant took notes recording body language and impressions, and operated the audio recorder.

Recruitment of participants for discussions and interviews stopped till “saturation” was reached, meaning there was no new information or themes emerge from the discussions and interviews.

In the RDS survey, participants completed a computer-assisted personal interview (CAPI) administered questionnaire. Questions included demographic information, sexual behaviours, HIV testing experience, drug use, experience of participating in HIV prevention programs. We also asked partner-by-partner sexual behaviour, condom use, and HIV status awareness questions for up to three male partners and two female partners within the prior 6 months. We asked three questions about participation of HIV prevention programs: if they received free condoms/lubricant; if they received free STIs examination; and if they received free VCT. The complete CAPI administered questionnaire was pilot tested among MSM volunteers in the real-life survey setting. Serological specimens collected from participants were tested for syphilis (rapid plasma regain (RPR) test, Shanghai Rongsheng, China) with confirmation of positive tests by the Treponema pallidum particle assay (TPPA) test (Fujirebio inc., Japan), and HIV-1 antibody (enzyme-linked immuno-sorbent assay (ELISA), Vironostika HIV Uni-Form plus O, bioMerieux, Holland) with confirmation by Western Blot confirmation (HIV Blot 2.2 WBTM, Genelabs Diagnostics).

### Data management and analysis

Data from the RDS survey were analysed to produce population point estimates using specific software Respondent-Driven Sampling Analysis Tool (RDSAT) version 5.6, which adjusted for personal network size and homophily in recruitment. The crude and RDSAT-adjusted univariate analyses were calculated for the characteristics of MSM in the sample. Any one who answered at least one “yes” for the three questions about participation was considered as “participation”. Bivariate associations between selected variables and program participation were conducted using RDSAT-generated weights on the outcome imported into in SAS version 9.1.

Variables significant at a level of P < 0.10 in bivariate analyses were considered candidates for multivariate models. Multivariate logistic regression models were constructed (using RDSAT-generated program participation weights) to select independent factors for program participation, while controlling for potential confounding factors. Both adjusted odds ratio (AOR) and CI were obtained for each explanatory variable in the final model. Since different programs were operating in Beijing (the Bill & Melinda Gates Foundation) and Chongqing (the Global Fund AIDS Program Round Five), and in order to find different patterns of participation of HIV prevention programs in two kinds of big cities, one is capital city in north China, and another is the biggest city in southwest China, separate analyses were conducted and compared for the two cities.

Using the expansion design, qualitative data were mined to answer questions derived from the quantitative analysis- notably, what were the reasons for engagement in HIV prevention programs and what were the barriers to participation. These findings are presented in order to provide context for the quantitative findings regarding HIV program coverage and exposure among MSM in Beijing and Chongqing.

Audio recordings of the focus group discussions and in-depth interviews were transcribed verbatim in Chinese and translated into English. The texts were entered into Atlas.ti 5.0. Data were reviewed for main themes and then coded for retrieval and analysis by an independent analyst. All texts were initially coded using a priori codes, and then data were coded again inductively based on findings from the data. Matrices were then created based on the data to help facilitate the comparison of text across different categories of informants.

### Protection of human subjects

The study was approved by the Committees for Human Research of the National Centre for AIDS/STD Control and Prevention of the China Centre for Disease Control and Prevention, Vanderbilt University and the University of California San Francisco.

## Results

### Equilibrium of the RDS sample

Recruitment chains progressed up to 15 waves in Chongqing and 18 waves in Beijing, depending on the particular branch. Equilibrium was reached in both cities on all key variables examined, including age, ethnicity, education, residence, marital status, income and living partner within 5 generations in the latest case.

### Demographic characteristics

A total of 498 MSM were recruited in Chongqing and 500 recruited in Beijing in the RDS survey. In Chongqing, almost 90% of participants were aged less than 34 years old, younger than those in Beijing (about 25% older than 35 years). Most participants in both cities were of Han ethnicity, unmarried (no significant difference detected between the two cities). More participants in Chongqing had higher education (P<0.001), were locally registered permanent residents (P<0.001), not full employed (P=0.004) and had a lower income (P = 0.026) than those in Beijing (Table 1).

**Table 1:** Demographic characteristics of recruited MSM in Chongqing and Beijing, 2010

	**Chongqing**		**Beijing**		**P (*χ***^**2**^**)**
	**Crude%(n)**	**Adjusted%**	**Crude%(n)**	**Adjusted%**	
**Age**					
<25	58.4 (291)	54.7	29.2 (146)	35.1	0.003
25~34	31.1 (155)	34.2	43.8 (219)	39.0	0.800
35~44	7.2 (36)	8.5	17.4 (87)	15.0	0.196
≥45	3.2 (16)	2.7	9.6 (48)	10.9	0.048
**Ethnicity**					
Han	97.4 (485)	97.4	93.8 (469)	93.6	0.457
Other	2.6(13)	2.6	6.2 (31)	6.4	
**Education**					
Secondary school and lower	8.2 (41)	7.6	25.4 (127)	30.1	<0.001
High school and higher	91. 8 (457)	92.4	74.6 (373)	69.9	
**Current marital status**					
Unmarried	92.0 (458)	88.2	84.6 (423)	79.3	0.223
Married	8.0 (40)	11.8	15.4 (77)	20.7	
**Living partner**					
Wife	6.0 (30)	8.6	5.8 (29)	7.0	0.959
Other female sexual partner	1.2 (6)	0.9	0.8 (4)	1.0	0.999
Boy friend	18.1(90)	18.1	21.8 (109)	19.0	0.996
Other male sexual partner	0.4 (2)	0.4	9.6 (48)	9.2	<0.001
Other/None	74.3 (370)	71.9	62.0 (310)	63.8	0.345
**Occupation**					
Full time	65.7 (327)	66.7	83.8 (419)	82.2	0.004
Others	34.3 (171)	33.3	16.2 (81)	17.8	
**Locally registered permanent Resident**					
Yes	78.5 (391)	69.5	19.6 (98)	15.9	<0.001
No	21.5 (107)	30.5	80.4 (402)	84.1	
**Income in the last year(RMB)**					
<3000	80.1 (399)	78.9	62.8 (314)	65.2	0.026
≥3000	19.9 (99)	21.1	37.2 (186)	34.8	
**Health insurance**					
Yes	54.2 (270)	53.3	55.0 (275)	52.2	0.997
No	45.8 (228)	46.7	45.0 (225)	47.8	

Twenty-four MSM in Beijing and 28 MSM in Chongqing participated in the FGDs. The age of participants ranged from 19 to 71 years old (mean age was 34 years in Beijing and 34.7 years in Chongqing), and the education level ranged from secondary school only to having a doctoral degree. Half (N=12) of all participants in Beijing and more than 90% (26 out of 28) of participants in Chongqing were locally registered permanent residents. Six participants in Beijing and six in Chongqing were HIV positive.

Thirty-four and 31 in-depth interviews (IDI) were conducted in Beijing and Chongqing, respectively. Participants included MSM (N=54) and non-MSM HIV prevention and care providers, outreach workers, or researchers (N=11). Of the 65 participants in the sample, about two thirds (66.2%) were between 25 and 44 years of age, about half (49.2%) had an education level of college or above. Twelve out of 34 in Beijing, and 25 out of 31 in Chongqing were locally registered permanent residents.

### Participation in HIV prevention programs

Adjusted analysis showed that in the past 12 months, about 40% of participants in Chongqing and two thirds of participants in Beijing received free condoms/lubricant (χ^2^test, P<0.001); about a quarter of participants in Chongqing and 45% in Beijing ever received free STIs examination (χ^2^test, P<0.001); about 37% of participants in Chongqing and 46% in Beijing received free voluntary HIV counselling and testing (χ^2^test, P=0.289). In summary, 56% of MSM in Chongqing and 75.1% in Beijing ever participated in at least one of the types of programs described above. (χ^2^test, P=0.001) (Table 2).

**Table 2:** Utilization of HIV prevention programs among MSM in Chongqing and Beijing, 2010

	**Chongqing 2010**		**Beijing 2010**		**P (*χ***^**2**^**)**
	**Adjusted%(n)**	**95%CI**	**Adjusted%(n)**	**95%CI**	
**Got free condoms/free lubricant in the past one year**					
Yes	39.3(238)	(33.9,46.6)	66.1(380)	(58.9,72.9)	<0.001
No	60.7(260)	(53.4,66.1)	33.9(120)	(27.1,41.1)	
**Got free STIs examination or treatment in the past one year**					
Yes	25.3(167)	(20.4,30.4)	45.0(296)	(38.0,52.4)	<0.001
No	74.7(331)	(69.6,79.6)	55.0(204)	(47.6,62.0)	
**Got free VCT in the past one year**					
Yes	37.1(228)	(31.3,43.6)	46.5(302)	(39.5,54.0)	0.289
No	62.9(270)	(56.4,68.7)	53.5(198)	(46.0,60.5)	
**Participated in any HIV prevention program**					
Yes	56.0(326)	(49.6,63.0)	75.1(411)	(68.9,81.3)	0.001
No	44.0(172)	(37.0,50.4)	24.9(89)	(18.7,31.1)	
Participated in any HIV prevention program (after standardization*)					
Yes	56.0(279)	-	65.6(328)	-	0.002
No	44.0(221)	-	34.4(172)	-	

*Direct standardization using age structure combining two cities.

Since age structures were different between the two cities, a standardized proportion was compared using combined age structure of the two cities (direct standardization). After standardization, 56% of MSM in Chongqing and 65.6% in Beijing ever participated in at least one of the types of programs described above. (χ^2^test, P=0.002) (Table 2).

Participants were asked where they most heard of the HIV prevention programs. The three most frequent answers raised by MSM in Chongqing were Internet (36.5%), friends/relatives (31.6%) and peers (22.6%). The three most frequent answers raised by MSM in Beijing were friends/relatives (50.6%), peers (34.1%) and sexual partner (11.1%).

### Factors associated with participation- Bivariate analysis

Weighted analyses were conducted separately because the two cities are not connected to each other via recruitment.

Bivariate analysis in Chongqing showed that MSM between 35 and 44 years of age were less likely to participate in HIV prevention programs (χ^2^test, P=0.001). Other factors positively associated with participation in HIV prevention programs include: Han ethnicity(χ^2^test, P=0.001), higher education level(χ^2^test, P=0.004), being unmarried(χ^2^test, P=0.023), living with boyfriend(χ^2^test, P=0.001), living in urban area(χ^2^test, P<0.001), bigger size of MSM social network (10 and above) (χ^2^test, P<0.001), fewer male partners in the past 6 months (less than 3) (χ^2^test, P=0.018), no female partner in the past 6 months(χ^2^test, P=0.040), no casual partner(χ^2^test, P<0.001), having talked about HIV status with partner(χ^2^test, P<0.001), knowing someone who is HIV positive(χ^2^test, P<0.001). However, 61.5% of MSM with a high perceived risk of acquiring HIV (vs. moderate, small and no risk perception) did not participate in HIV prevention programs (χ^2^test, P=0.001) (Table 3).

**Table 3:** Factors associated participation in HIV prevention program-Bivariate weighted analysis (Chongqing vs Beijing, 2010)

	**Chongqing**			**Beijing**		
	**Participated**			**Participated**		
	**Yes N (%)**	**No N (%)**	**P (*χ***^**2**^**)**	**Yes N (%)**	**No N (%)**	**P (*χ***^**2**^**)**
**Age**						
<25	159(56.9)	109(49.9)	0.001	126(33.5)	43(34.9)	0.982
25~34	104(37.4)	74(33.8)		145(38.6)	49(39.0)	
35~44	10(3.6)	28(12.8)		58(15.5)	18(14.7)	
≥45	6(2.1)	8(3.5)		47(12.4)	14(11.4)	
**Ethnicity**						
Han	277(99.4)	207(94.4)	0.001	345(91.9)	124(99.4)	0.003
Other	2(0.6)	12(5.6)		30(8.1)	1(0.6)	
**Education**						
Secondary school and lower	12(4.4)	24(11.1)	0.004	114(30.3)	33(26.8)	0.456
High school and higher	267(95.6)	195(88.9)		262(69.7)	91(73.2)	
**Current marital status**						
Unmarried	255(91.3)	186(84.8)	0.023	306(81.5)	91(73.6)	0.057
Married	24(8.7)	33(15.2)		69(18.5)	33(26.4)	
**Living partner**						
Wife	19(6.7)	24(10.7)	0.001	24(6.4)	13(10.1)	0.001
Other female sexual partner	1(0.3)	3(1.6)		4(1.0)	1(0.8)	
Boy friend	71(25.3)	25(11.6)		88(23.5)	8(6.7)	
Other male sexual partner	2(0.6)	0(0.1)		30(8.0)	16(13.2)	
Other/None	187(67.0)	166(76.0)		230(61.2)	86(69.1)	
**Living place**						
Urban area	269(96.4)	186(84.9)	<0.001	325(86.5)	109(87.3)	0.801
Other	10(3.6)	33(15.1)		51(13.5)	16(12.7)	
**Income in the last year(RMB)**						
<3000	217(77.6)	174(79.3)	0.651	264(70.3)	62(49.6)	<0.001
≥3000	63(22.4)	45(20.7)		112(29.7)	63(50.4)	
**Size of MSM social network**						
10 and above	67(24.1)	25(11.6)	<0.001	119(31.7)	22(17.4)	0.002
<10	212(75.9)	194(88.4)		257(68.3)	103(82.6)	
**Number of male partners in the past 6 months**						
3 & above	42(15.2)	52(23.6)	0.018	140(37.2)	28(22.2)	0.002
Less than 3	237(84.2)	167(76.4)		236(62.8)	97(77.8)	
**Had female partner in the last 6 months**						
Yes	30(10.9)	38(17.2)	0.040	60(15.9)	21(17.2)	0.721
No	249(89.1)	181(82.8)		316(84.1)	103(82.8)	
**Had male casual partners**						
Yes	112(40.3)	125(57.1)	<0.001	225(59.9)	71(56.9)	0.552
No	167(59.7)	94(42.9)		151(40.1)	54(43.1)	
**Talked about HIV status with partner**						
Yes	191(68.4)	102(46.4)	<0.001	273(72.8)	63(50.5)	<0.001
No	88(31.6)	117(53.6)		102(27.2)	42(49.5)	
**Risk perception**						
High	125(44.6)	135(61.5)	0.001	33(8.9)	5(4.3)	0.092
Moderate	76(27.4)	39(17.9)		80(21.3)	21(16.5)	
Small/None	78(28.0)	45(20.6)		262(69.8)	98(79.2)	
**Know Sb. HIV+**						
Yes	57(20.4)	19(8.6)	<0.001	43(11.4)	14(11.1)	0.945
No	222(79.6)	200(91.4)		333(88.6)	110(88.9)	
**Syphilis**						
Positive	5(4.9)	9(3.9)	0.604	53(14.1)	9(7.3)	0.046
Negative	266(95.1)	210(96.1)		323(85.9)	115(92.7)	

In Beijing, factors positively associated with participation in HIV prevention programs include: ethnicity of minority (other than Han)(χ^2^test, P=0.003), living with boyfriend(χ^2^test, P=0.001), less income (<3000 per month) (χ^2^test, P<0.001), bigger size of MSM social network (10 and above) (χ^2^test, P=0.002), more partners in the past 6 months (more than 3) (χ^2^test, P=0.002), having talked about HIV status with partner(χ^2^test, P<0.001), syphilis positive(χ^2^test, P=0.046). Different from participants in Chongqing, MSM with high risk perception (vs. moderate, small and no risk perception) were more likely to participate in HIV prevention programs (χ^2^test, P=0.092) (Table 3).

Other characteristics such as having casual partners, inconsistent use of condoms were not associated with participation of HIV prevention programs in both cities.

### Factors associated with participation- multivariate analysis

Weighted logistic regression was conducted for the two cities to examine factors associated with participation in HIV prevention programs, controlling for confounders. In Chongqing, MSM with age of 35–44 years and high HIV risk perception were less likely to participate in HIV prevention programs, with odds ratios of 0.29 (95%CI: 0.13 to 0.67) and 0.63(95%CI: 0.42 to 0.93), respectively. Participants with the following characteristics were more likely to participate in HIV prevention programs: Han ethnicity (OR, 95%CI: 13.0, 2.37 to 71.8), living with boyfriend (OR, 95%CI: 1.99, 1.15 to 3.43), living in urban area (OR, 95%CI: 4.12, 1.85 to 9.19), bigger size of MSM social network (OR, 95%CI: 2.13, 1.22 to 3.73), having talked about HIV status with partners (OR, 95%CI: 2.72, 1.81 to 4.07), knowing someone who is HIV positive (OR, 95%CI: 2.21, 1.17 to 4.17) (Table 4).

**Table 4:** Factors associated with participation of HIV prevention programs in Chongqing, 2010 (Weighted Logistic Regression)

	***β***	***S*.*E*.**	***Waldχ***^***2***^	***P***	***OR***	***95*%*CI***
Aged between 35 &44	−1.223	0.422	8.416	0.004	0.29	0.13~0.67
Ethnicity (Han)	2.562	0.873	8.612	0.003	13.0	2.34~71.8
Living with boyfriend	0.688	0.278	6.111	0.013	1.99	1.15~3.43
Living in urban area	1.416	0.409	11.972	0.001	4.12	1.85~9.19
Knowing more gays in city(≥10)	0.756	0.286	6.979	0.008	2.13	1.22~3.73
Talked about HIV status with partner	0.999	0.206	23.431	<.0001	2.72	1.81~4.07
High HIV risk perception	−0.471	0.204	5.319	0.021	0.63	0.42~0.93
Knowing Sb. HIV +	0.792	0.324	5.960	0.015	2.21	1.17~4.17

Different from those in Chongqing, MSM in Beijing with Han ethnicity were less likely to participate in HIV prevention programs, with odds ratio of 0.07 (95%CI: 0.01 to 0.75). Participants in Beijing with the following characteristics were more likely to participate in HIV prevention programs: living with boyfriend (OR, 95%CI: 3.92, 1.80 to 8.53), lower income (OR, 95%CI: 3.38, 2.11 to 5.42), bigger size of MSM social network (OR, 95%CI: 2.26, 1.28 to 3.96), more partners in the past 6 months (OR, 95%CI: 1.74, 1.04 to 2.92) and having talked about HIV status with partners (OR, 95%CI: 2.60, 1.62 to 4.18) (Table 5).

**Table 5:** Factors associated with participation of HIV prevention programs in Beijing, 2010 (Weighted Logistic Regression)

	***β***	***S*.*E*.**	***Waldχ***^***2***^	***P***	***OR***	***95*%*CI***
Ethnicity (Han)	−2.635	1.196	4.854	0.028	0.07	0.01~0.75
Living with boyfriend	1.365	0.397	11.818	0.001	3.92	1.80~8.53
Lower income(<3000RMB)	1.217	0.241	25.428	<.0001	3.38	2.11~5.42
Knowing more gays in city(≥10)	0.813	0.288	7.976	0.005	2.26	1.28~3.96
More partners last 6 mo(≥3)	0.555	0.264	4.404	0.036	1.74	1.04~2.92
Talked about HIV status with partner	0.956	0.242	15.564	<.0001	2.60	1.62~4.18

### Barriers to participation among Chinese MSM

Using the qualitative data, we found that a major barrier to participation in HIV prevention activities was being out as gay in their community; thus they likely do not share any of the characteristics of those who are more likely to participate such as a large MSM network or having a boyfriend. This group of MSM is characterized below.

"Those who do not want to participate in AIDS programs are those have family. They do not want to acknowledge that they are gay. (In-depth interview, Chongqing, 41 year-old MSM, secondary school, security guard)"

In addition to being openly gay and being low income, MSM reported a number of logistic factors that affect their desire to participate in HIV Prevention programs. For example, distance to testing sites and ostensibly, other HIV prevention programs, was a challenge.

"(Q:why did you not go?) Testing site is not convenient, it’s too far. (Q: there are many places you can take the test) But I do not know where they are. (In-depth interview, Beijing, 34 year-old MSM, secondary school, self-employed)"

The intervals between activities was also important to MSM; i.e. that programs that occurred too often were undesirable.

"Sometimes it’s no use to organize prevention activities too frequently, do it with some intervals. (In-depth interview, Beijing, 42 year-old MSM, secondary school, volunteer)"

Though many participants mentioned the internet as a tool for reaching and engaging MSM, interventions on the internet were not without problems, some MSM were afraid of being “cheated”.

"Another reason why MSM did not participated in programs is that, if you ask me to come for the program, and I don’t know you, I would be afraid of being cheated: what if it ‘s a trap? There are so many cheaters on the internet. If you are someone that I am familiar with and I have time, then I may come. Otherwise……I am not coming(In-depth interview, Chongqing, 26 year-old MSM, secondary school, salesclerk)."

A number of participants expressed distrust of the HIV prevention programs, which affected their desire to participate. Specifically, concerns over the quality of data coming from some of the HIV prevention programs were mentioned and MSM discussed a need for evaluation activities to determine which programs were reaching unduplicated clients.

"The organizer did not have a specific objective or target population for the activities organized. Someone come today, someone come tomorrow, and someone come over and over again. Wherever had AIDS-related activities, they went. (Q:Do they have a identification system?). No such a system. Many testings were conducted, but testees were almost the same people. In statistics, the sample was not representative. (In-depth interview, Beijing, 37 year-old MSM, college, accountant)"

Others described poorly designed HIV prevention programs that were seen as superficial and repetitive. Poorly delivered programs unfortunately decreased the reputation of these organizations among MSM, and decreased the likelihood that MSM would engage with these services in the future.

"There were too many duplicates. I participated in many education activities, the content were all about how to prevent disease, how to use condoms. (In-depth interview, Beijing, 27 year-old MSM, high school, self-employed)"

"Take distributing condoms as an example, they just sent out condoms and left, there was totally no communication. It has very limited effect because we can always buy condoms in drug store. (In-depth interview, Chongqing, 46 year-old MSM, college, self-employed)"

Some participants expressed concern that the social networks that were being formed were not managed properly and instead of creating safe environments, created riskier ones. For example, one participant described how a volunteer became infected with HIV after meeting participants through the HIV prevention program.

"Some volunteers in NGOs got infected. I heard that Mr. A, working as volunteer, had many partners and was infected. Some MSM were confused and became distrust NGOs. The capacity of NGOs needs to be improved. Some NGOs just exist for finishing program. (In-depth interview, Chongqing, 25 year-old MSM, college, self-employed)"

Another participant described how the opportunity was lost to utilize social networks positively by diffusing effective HIV prevention education messages through these growing networks.

"More education is needed. For HIV testing, it would be better if testing place is convenient, or you have more testing places, and confidential. …Education should be conducted routinely. Because MSM make new friends, you could ask them if they disseminate AIDS knowledge to their new friends, collect their suggestions on these activities. It’s good for controlling AIDS. Yes, routine education. (In-depth interview, Chongqing, 36 year-old MSM, high school, self-employed)."

A number of cultural reasons were given as to why MSM did not participate in HIV prevention programs, most of which were related to HIV and gay-related stigma. A number of MSM reported that they would not participate in HIV prevention programs due to the assumption that HIV is related to being gay, thus they would be “outted” as gay by participating. MSM were also fearful of being labelled as HIV positive if they were to participate.

"The first reason, your colleagues would know you are a gay. Another one, other gays would suspect that you are infected with HIV so you participate in those programs or work as a volunteer. (In-depth interview, Beijing, 37 year-old MSM, college, accountant)"

Others reported more simply being shy or having low risk perception based on “feeling healthy” and/or believing they did not engage in high risk behaviours.

### Facilitators to participation

A number of positive aspects of HIV prevention programs were also described, which may be facilitators to participation for Chinese MSM. HIV prevention programs were seen as good if they were easily accessible, confidential, small scale, and at convenient times. Perhaps most importantly for low income MSM, money or other free goods were a major draw. Qualitative data from Beijing showed that the need for money greatly superseded concerns about HIV, which may explain the quantitative finding that low income MSM in Beijing are more likely to participate in HIV prevention programs.

"There is a tendency in Beijing: If you give me money or provide meals in prevention activities, I will go. Otherwise I won’t go. Free HIV testing? No.(Q:Don’t they care about if they are infected?)But they care more about if there is money. Because many MSM are lazy: when they have money, they can eat better. Testing is not important for them. (In-depth interview, Beijing, 26 year-old MSM, high school, volunteer)"

Some MSM passively participated in HIV prevention programs, for example, by attending parties organized by NGOs where they were exposed to watching education shows, taking HIV tests, condom and lubricants, participating for gifts or meals, participating to make friends, etc. Therefore, gifts, good incentives and meals may attract more MSM to participate in HIV prevention activities.However, some MSM were concerned that programs induced participation using incentives, and some volunteers participated in interventions solely to earn money.

There was some discussion among participants about the importance of the internet both for reaching isolated MSM and for delivery of programs. Participants thought that a particularly useful use of the internet would be for HIV prevention programs to use it to reach MSM who were not out in their community.

"On the other hand, they might think that the HIV/AIDS knowledge can be accessed through educational materials in bathhouses and bars, or from the Internet. (In-depth interview, Chongqing, 41 year-old MSM, secondary school, security guard)"

An innovative idea proposed by a participant was to utilize the internet for interventions and to get new ideas for marketing programs from participants themselves was also raised by MSM.

"Education should not be limited in venues. Internet is also important. There are too few people in bars, and they are often constant customers. Distributing IEC materials by MSM volunteers would be better. They can disseminate to their friends, and the friends also have their own friends. (In-depth interview, Chongqing, 29 year-old MSM, secondary school, office clerk)."

HIV prevention programs that were perceived to be good were those that MSM thought really cared about their community, were well organized, and could recruit more MSM. Despite high marks for some programs, many MSM reported poorly designed and delivered programs. An important programmatic recommendation by participants was to have medical experts and other small scale activities, which may work best for HIV prevention with Chinese MSM

"It would be better if you can invite some medical experts to talk about treatment of AIDS. I like this kind of activities. …Small scale activities is better than big scale. Big scale activities is hard to organize and not convenient. (In-depth interview, Beijing, 42 year-old MSM, secondary school, volunteer)"

## Discussion

Our study shows that, over half of participants in Chongqing and three fourth of participants in Beijing participated in at least one HIV prevention program including receiving free condoms/lubricants, free VCT, and free STIs examination in the past 12 months. For HIV testing, about 37% of participants in Chongqing and 46% in Beijing got free voluntary HIV counselling and testing. MSM participated in a variety of HIV prevention programs, in addition to condom distribution, STIs and VCT services. More MSM actively participated in the reduction of HIV transmission working as volunteer. Utilization of HIV prevention programs in our results is higher than other studies. For example, in a study in one of the two cities in our study, Beijing, conducted in 2002, Choi found that only 18% of recruited MSM had ever tested for HIV before the study, much lower than current study in 2010 [26]. The increase of coverage and variety of HIV prevention programs may be partly due to the fact that the Chinese Government started to pay attention to HIV prevention among MSM in the last a few years [5]. More and more domestic and international funding has been invested in HIV prevention among this high risk group. For example, in addition to government investment, the Global Fund AIDS Program Round Five in Chongqing and the Bill & Melinda Gates Foundation in Beijing both conducted many prevention programs among MSM and the accessibility of HIV prevention programs has greatly improved [27,28]. However, the overall participation in HIV prevention programs remains low among MSM: no program coverage exceeded 50% in either cities, except for condom distribution in Beijing. This indicates that more effort is needed to increase the coverage of HIV prevention programs among MSM.

There have been studies exploring factors of and barriers to utilization of HIV prevention programs among MSM. Choi (2006) found that being older, having a college degree, being more “out” (disclosing MSM activities to others), being recruited through social networks, and having a lifetime history of sexually transmitted diseases were statistically significant independent correlates of having been tested for HIV. The most common reasons for not testing were perceived low risk of HIV infection, not knowing the location of test sites, fear of positive test results, fear of people learning about his homosexuality, and fear of breach of confidentiality about test results [26]. In Li’s study in nine cities in Hubei, Anhui and Hebei Provinces (2009), knowing VCT, having been mobilized to accepted HIV testing by doctors, worrying about being infected by HIV, etc., were promoting factors of VCT used among MSM, while thinking local VCT service unnecessary and not knowing local situation of HIV prevalence were main obstacle factors [29]. In India (2009), Thomas and colleagues found that MSM reporting unprotected anal sex in the prior 3 months were less likely to have participated in an HIV prevention intervention. MSM who were older, those with higher educational attainment, and MSM who were “out” about having sex with other men were more likely to have reported participating in an HIV prevention program [23]. Behel (2008) also found ethnicity as a factor of receiving prevention services: compared with white MSM, black and Hispanic MSM were more likely to believe that HIV prevention services are important, and were more likely to receive prevention services at their health care providers [30]. Miller (2003), in his study found that MSM did not like HIV prevention programs that did not care about them and they wanted providers do something other than throw condoms at men [31]. Respondents in Seal’s study (2000) noted a need for comprehensive HIV prevention programs that addressed issues related to dating and intimacy, sexuality and arousal, drugs and alcohol, self-esteem and self-worth, abuse and coercion, and sexual identity. They also emphasized the importance of keeping programs confidential, fun, comfortable, accepting and open to all young MSM [32]. Courtenay-Quirk (2003) reported that topics specific to HIV prevention were of interest to most MSM, but the level of interest was generally lower than for other topics [33].

In our study, factors associated with participation in HIV prevention programs were not only a bit different from previous studies, but also between the two cities. Ethnic Han MSM in Chongqing were more likely to participate in HIV prevention programs, but ethnic Han MSM in Beijing were less likely to participate. Possible reason might be that ethnic minorities (other than Han) in Beijing have lower income and incentives of the programs are attractive to them, whereas in Chongqing, the survey was conducted in urban area where ethnic minorities live sparsely and have limited access to HIV prevention programs. This finding might also be caused by chance since the number of non-Han people in both cities was small. Living with boyfriend, bigger size of MSM social network, and having talked about HIV status with partner were associated with participation in HIV prevention programs in both cities. Living in urban area and knowing someone who is HIV positive were unique factors in Chongqing and lower income, more partners in the past 6 months were unique factors in Beijing. This indicates that future efforts to increase coverage of HIV prevention programs should focus on MSM with different characteristics in the two cities. For example, in Chongqing, HIV prevention program should target on those MSM who are ethnic minorities, living in rural area, and with lower perception of AIDS, whereas in Beijing, MSM who are ethnic Han, with lower income and more partners should be paid more attention. In both cities, interaction and communication between MSM could be helpful, for example, using peer education.

In our study, HIV and syphilis status were not found to be related to participation of HIV programs, although syphilis positive was positively associated in bivariate analysis of participants in Beijing. This might suggest that implementation of HIV prevention programs could be effective for any MSM, no matter if they are infected. Another possible explanation might be potential confounders that were not identified.

Our qualitative data on MSM's perception of good and poor HIV prevention programs and reasons of not participating in programs are also helpful to develop future prevention programs in the two cities and hopefully in other cities in China. Logistic considerations for future HIV prevention programs to consider are making programs easily accessible, confidential, small scale, at convenient times with gifts, good incentives and meals. Participants also suggested spacing out the HIV prevention programs so as to not make them always available and to utilize the internet for interventions. Service and delivery related suggestions from participants were to have cutting edge educational topics and delivery methods that were not repeated. A couple examples provided by participants were to diffuse information through newly formed social networks as a positive way to engage the community and to use gay men who were celebrities as peer educators. Participants also discussed the need for monitoring and evaluation activities to help provide regular feedback, modify program content, and collect outcomes data. Such data may also help organizations provide better content, which may increase trust between MSM who were previously disappointed and offended by previous services and providers. Cultural issues may be more difficult to overcome but based on these data, need to be addressed. HIV and gay-related stigma are important barriers to engagement in HIV prevention programs especially among those who most may need to be engaged in HIV prevention activities.

Our findings are subject to several limitations. First, although we identified factors associated with participation in HIV prevention programs, some of them are susceptible of time ambiguity with participation because of the cross-sectional nature of the survey. That is, some factors (events) maybe have occurred after participation in HIV prevention programs, for example, knowing someone who is HIV positive could have occurred due to the HIV prevention program and not been the catalyst for initial participation. Participation of HIV prevention programs might also changed their risk perception. Second, our data were drawn from only two municipalities in China and do not represent the whole country. Even for the two cities, the recruited sample of MSM may not be representative, even using RDS adjustments. MSM is a hard-to-reach population all over the world, there may always be some MSM who will never participate in research. Thus, our estimation of proportion of MSM who participated in HIV prevention programs may be over estimated. Third, report bias on sensitive questions may still have occurred although we used a computer-assisted self-administered techniques to overcome this bias.

Despite the limitations of our study, we believe that the results of our study are helpful to understand MSM’s participation of HIV prevention programs and to guide development of future prevention programs among this population in China. Based on our findings, we suggest that: 1) HIV prevention programs targeting MSM should be vigilant in meeting specific needs of the population, for example, programs must guarantee confidentiality, provide more topics MSM are interested in and that will encourage attendance, be easily accessible (i.e. in areas already attended by MSM and not at centrally located offices of CDC), high quality of services and more communications; 2) go beyond traditional HIV prevention programming (i.e. VCT, IEC) and develop innovative programs that have been proven effective and attractive, for example, popular opinion leader intervention [31], Internet-based interventions [34], mobilizing gay men to support each other about safer sex to reduce risk of HIV infection [35]; 3) eliminate structural barriers to increase coverage of HIV prevention programs, as suggested by Sumartojo [36]. HIV-prevention research targeting MSM needs to focus on contextual and structural factors [37], for example, reduce discrimination towards MSM and AIDS patients, and develop laws to protect the rights of MSM.

## Conclusions

This mixed-method study integrating qualitative findings with quantitative factors shows that in a rigorous sample of MSM recruited through RDS, over half of participants in Chongqing and three fourth of participants in Beijing participated in at least one HIV prevention program in the past 12 months. However, there is much room for improvement in reaching MSM in China. Factors associated with participation in HIV prevention programs and reasons of not participating in programs are discussed. HIV prevention programs targeting MSM in China may need to be more comprehensive and incorporate the cultural, logistic and HIV-related needs of the population in order to effectively reach and affect this population’s risk for HIV. The results of this study will be helpful to develop future prevention programs in the two cities and hopefully in other cities in China.

## Competing interests

The authors declare that they have no financial or non-financial competing interests.

## Authors’ contributions

WM carried out data analysis, drafted the manuscript and participated in design of the study; ECW assisted with manuscript writing and participated in the interpretation of results and discussion; HFR WM and YJ conceived and designed the study, and participated in the interpretation of results and discussion; JS YS YR and YX designed and coordinated the study, and participated in the interpretation of results and discussion; HL XD RL XM DX JX XH LF SF and XL coordinated the study, and participated in the collection, analysis and interpretation of data. All authors read and approved the final manuscript.

## Pre-publication history

The pre-publication history for this paper can be accessed here:

http://www.biomedcentral.com/1471-2458/12/847/prepub
